# Analysis of factors affecting the construction duration of public health emergency medical facilities

**DOI:** 10.3389/fpubh.2023.1162804

**Published:** 2023-05-04

**Authors:** Wenchao Zhao, Haibo He

**Affiliations:** ^1^Business School, Sichuan University, Chengdu, China; ^2^College of Civil Engineering and Architecture, Xinjiang University, Urumqi, China

**Keywords:** COVID-19, emergency-medical-facilities, construction duration, fsQCA method, path-configurations

## Abstract

**Objectives:**

This study explores the factors influencing the construction duration of public health emergency medical facilities and the ways in which they can be enhanced.

**Methods:**

Combining 30 relevant emergency medical facility construction cases in different cities in China from 2020 to 2021, seven condition variables and an outcome variable were selected, and necessary and sufficient condition analyses of duration influence factors were conducted using the fsQCA method.

**Results:**

The consistency of seven condition variables was <0.9, which shows that the construction period of public health emergency medical facilities is not independently affected by a single condition variable but by multiple influencing factors. The solution consistency value of the path configurations was 0.905, indicating that four path configurations were sufficient for the outcome variables. The solution coverage of the four path configurations was 0.637, indicating that they covered ~63.7% of the public health emergency medical facility cases.

**Conclusion:**

To reduce the construction duration, the construction of emergency medical facilities should focus on planning and design, the selection of an appropriate form of construction, the reasonable deployment of resources, and the vigorous adoption of information technology.

## Introduction

The COVID-19 outbreak in early 2020 spread rapidly worldwide, resulting in an alarming increase in infections and deaths across 216 countries and territories (1–4). Healthcare systems worldwide are being overwhelmed by a lack of treatment experience (5, 6), and the increased demand for beds and medical equipment due to the increase in the number of patients has not been effectively met (7, 8). The epidemic has had a significant impact on the orderly development of cities and societies (9), and many countries have been able to increase healthcare capacity and restrict the spread of COVID-19 outbreaks in a short period by quickly building emergency medical facilities (10–12).

Taking China as an example, the Chinese government drew lessons from the 2003 SARS outbreak in Xiaotangshan (13, 14) and developed a significant number of public health emergency medical facilities to treat coronavirus-infected patients (14–17). There are three types of public health emergency medical facilities: (1) freshly constructed emergency hospitals utilizing modular and standardized containers, such as Leishenshan Hospital and Huoshenshan Hospital; (2) emergency hospitals created by renovating and expanding existing hospitals; and (3) shelter hospitals created by renovating major public facilities, such as stadiums and exposition halls. For example, emergency hospitals are mostly used to admit severely ill patients, whereas shelter hospitals primarily isolate and treat moderately ill patients (18).

The construction and use of emergency medical facilities have alleviated chronic bed shortages (10, 14), effectively reducing the risk of COVID-19 transmission in households and communities (12), reducing the number of new infections and the proportion of seriously ill patients, and playing a critical role in controlling the spread of the epidemic and improving the COVID-19 cure rate. Emergency medical facilities require government, enterprises, and other parties to quickly coordinate human, financial, and material resources (19, 20) to rapidly undertake the huge planning, design, and construction workload. Many hospitals have achieved good results using the engineering procurement construction (EPC) management mode, design, and existing resources (21). However, numerous issues require reflection and improvement during construction (22), such as material waste, excess labor, the potential risk of epidemic transmission, improper site selection, and management confusion. These issues result in the waste of human, material, and financial resources and the failure to complete the construction on time and affect the overall epidemic prevention and control situation.

In urgent situations and resource-constrained emergencies, it is essential to ensure the rapid construction of public health emergency medical facilities. Chan et al. (23) created a framework of engineering project effect variables and analyzed how various elements affect the construction duration. Chan and Kumaraswamy (24) classified the duration influencing factors into six categories based on a summary of previous study findings and a large number of engineering projects: Project-Scope, Project-Complexity, Material-Condition, Manage-Attributes, Environment, and Other-Factors. Xu and Sun (25) used this framework to analyze the factors affecting the duration of post-Wenchuan earthquake reconstruction in 2008. There is currently a lack of systematic guiding theory for constructing public health emergency medical facilities. The literature and research on the factors influencing the construction duration of public health emergency medical facilities are insufficient. Therefore, it is necessary to conduct this research. As such, this study takes the construction duration as the main research object, explores the factors influencing the construction duration of public health emergency medical facilities and the improvement path, and provides some reference value policy suggestions for their construction.

## Materials and methods

### Data

To ensure the study's correctness, the author acquired 285 relevant data points (Table 1) for use in the study from 2020 to 2021, and 271 valid data points were obtained after analysis and screening.

**Table 1:** Data type and sources (China, 2021).

**Data type**	**Sources**	**Number**	**Effective number**
Research papers	Google, CNKI	25	24
Construction news reports	CCTV news, mainstream media	65	60
Questionnaires	Survey interviews with a construction person	195	187

The study cases had to meet the following conditions: (1) the cases were widely publicized. We can gain a more comprehensive understanding of public health emergency medical facilities by selecting cases reported in official media and newspapers, which are well known to the general public. (2) The correctness of the case is assured by the precision of the project's unique data and the concurrent management of the construction phase. (3) The data sources are various, ensuring that the study's coverage and correctness match the actual scenario.

### Method selection

QCA focuses on examining the causal link between causal conditions and outcomes, which results in more than one combination of causal conditions (26). The combination of causal conditions is necessary for obtaining the result, and the adequate configuration of the causal conditions under various situations is thoroughly studied. Varied path configurations of causal conditions have different effects on the findings, but they all have the same outcome (27).

This is a case study with a modest sample size of research cases. The QCA technique was chosen to account for the group effects among numerous condition variables and the causal complexity and interdependence among variables, as well as to investigate the joint effects and group linkages of many factors from different viewpoints. FSQCA contains both qualitative and quantitative features, making it more appropriate for this study.

### Variable selection

From Ragin (28), it is known that the number of variables in QCA cases with small to medium sample sizes should be 4–7. To meet the variable complexity of the actual construction situation, the number of condition variables was selected as seven.

**Figure 1:**
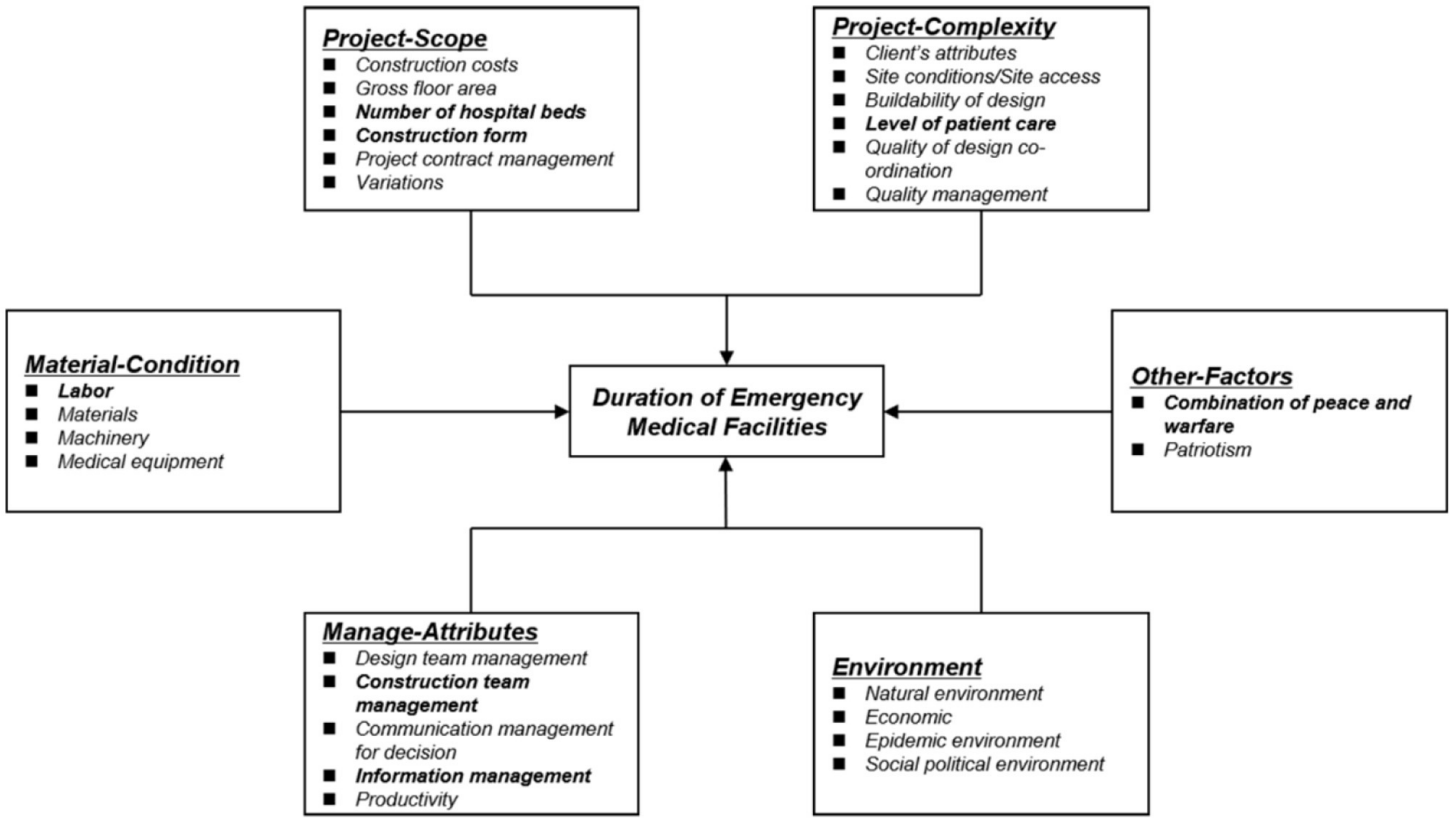
Factors affecting the duration of emergency medical facilities (China, 2021). The bolded text indicates the seven selected conditional variables.

This study identified a preliminary framework based on Chan and Scott's project management framework, considering the characteristics of public health emergency medical facilities. The qualitative analysis software NVivo was then used to analyze high-frequency words from 271 research papers, news report texts, and questionnaires, which allowed the identification of the main factors influencing the construction duration of public health emergency medical facilities (Figure 1). In conjunction with the research objectives, the outcome variable was set as the construction duration of the emergency medical facility, and the condition variables were determined by the results of the qualitative data analysis.

### Variable assignment

The outcome variable (construction duration) and condition variables (number of hospital beds, labor, construction team management, etc.) were assigned values according to actual values. Information management was assigned using the three-value method. The binary method was used to assign values to the level of patient care and the combination of peace and warfare. To simplify the calculation and explanation, NHB, CF, LBR, CTM, IM, LPC, CPAW, and DEMF were used to represent the variables number of hospital beds, construction form, labor, construction team management, information management, level of patient care, combination of peace and warfare, and duration of emergency medical facilities, respectively. The results of the variable assignment are shown in Table 2.

**Table 2:** Assignment values of variables and qualitative breakpoints (China, 2020–2021).

**Cases**	**Provinces**	**Care level**	**Numeration**	**NHB**	**CF**	**LBR**	**CTM**	**IM**	**LPC**	**CPAW**	**DEMF**
Chengdu Public Medical Center	Sichuan	Severe	Case 1	370	0.25	604	75	0.5	0	0	10
Huoshenshan Hospital	Hubei	Severe	Case 2	1,000	0.25	6,500	800	1	0	1	10
Leishenshan Hospital	Hubei	Severe	Case 3	1,600	0.25	8,000	1,000	1	0	1	12
Nanjing Public Health Medical Center	Nanjing	Severe	Case 4	396	0	2,000	200	1	0	0	12
Ren City Emergency Hospital	Shandong	Severe	Case 5	666	0.75	4,000	443	1	0	0	5
Xiaotangshan Emergency Hospital	Beijing	Severe	Case 6	1,600	0.25	5,000	500	1	0	1	53
Xi'an Public Health Center	Shanxi	Severe	Case 7	475	0.25	1,800	300	1	0	0	13
Shanghai Public Medical Project	Shanghai	Severe	Case 8	200	0.25	1,300	150	1	0	0	22
Guiyang Health Treatment Center	Guizhou	Severe	Case 9	404	0.25	1,200	100	0.5	0	0	20
Changsha Health Treatment Center	Hunan	Severe	Case 10	300	0.75	400	45	1	0	0	3
Qiboshan Hospital	Henan	Severe	Case 11	287	0.75	1,200	200	0.5	0	0	10
Xiaogan Southeast Hospital	Hubei	Severe	Case 12	375	0.75	40	10	0	1	0	3
Nanning Autonomous Region People's Hospital	Guangxi	Severe	Case 13	100	0.75	200	30	1	0	0	7
Chengdu Sancha Central Hospital	Sichuan	Mild	Case 14	26	0	110	8	0.5	1	1	8
Wuhan Living Room Shelter Hospital	Hubei	Mild	Case 15	1,461	1	600	90	0	1	1	3
Wuchang Shelter Hospital	Hubei	Mild	Case 16	700	1	150	30	0	1	1	2
Wuhan Sports Center Shelter Hospital	Hubei	Mild	Case 17	1,100	1	78	20	0	1	1	1
Hankou Shelter Hospital	Hubei	Mild	Case 18	2,000	1	480	50	1	1	1	7
Long March Shelter Hospital	Hubei	Mild	Case 19	1,200	1	120	30	1	1	1	2
Dun Kou Shelter Hospital	Hubei	Mild	Case 20	990	1	500	45	0	1	1	3
Jianghan International Center Shelter Hospital	Hubei	Mild	Case 21	1,600	1	700	150	0	1	1	2
New Town Shelter Hospital	Hubei	Mild	Case 22	3,500	1	2,000	350	1	1	1	3
Huangpi Shelter Hospital	Hubei	Mild	Case 23	300	1	300	75	0	1	1	1
Xiamen Airport Epidemic Emergency Project	Fujian	Mild	Case 24	400	0.25	1,360	52	0	1	1	3
Hubei Provincial Party-School Shelter Hospital	Hubei	Mild	Case 25	932	1	800	150	0	1	1	8
Xinzhou Shelter Hospital	Hubei	Mild	Case 26	200	1	150	20	0	1	1	2
Chengdu West District Isolation Hospital	Sichuan	Mild	Case 27	200	0.25	254	21	0	1	1	7
Wuhan City Vocational College Isolation Point	Hubei	Mild	Case 28	1,500	1	250	40	0	1	1	3
Hanyang International Center Shelter Hospital	Hubei	Mild	Case 29	1,000	1	200	35	0	1	1	1
Wuhan Convention Center Shelter Hospital	Hubei	Mild	Case 30	1,000	1	230	50	0	1	1	1
Qualitative breakpoints					
**Variables**	**Specific variables**	**Set target**	**Qualitative anchors for variables**		
			**Full non-membership (5%)**	**Crossover point (50%)**	**Full membership (95%)**
Causal conditions	NHB	Few	1,840	683	145
	LBR	Many	92	550	5,825
	CTM	Many	15	64	665
Outcome	DEMF (severe)	Short	21	10	4
	DEMF (mild)	Short	8	3	1

NHB, number of hospital beds; CF, construction form; LBR, labor; CTM, construction team management; IM, information management; LPC, level of patient care; CPAW, combination of peace and warfare (an isolation hospital in the epidemic period and a regular hospital in the post-epidemic era); DEMF, duration of emergency medical facilities.

### Data calibration

The outcome and condition variables must be calibrated into fuzzy sets with values ranging from 0 to 1 (29). Three meaningful thresholds are usually set: 0.95, 0.05, and 0.5, with 0.95 representing the threshold for full membership, 0.05 for full non-membership, and 0.5 for the crossover point (30, 31). The set target of the construction duration of the outcome variable is short, and the breakpoint value of complete affiliation is set to a shorter duration. Considering that there is a significant difference in the amount of medical facility work for treating seriously ill patients and treating lightly ill patients, the breakpoints are set separately. Under the premise of ensuring a shorter duration, the condition variables number of hospital beds, labor, and construction team management are set according to the principle of higher affiliation.

## Results

This study used fsQCA v3.0 software to conduct univariate necessity analysis and sufficient analysis of condition and outcome variables.

### Univariate necessity analysis

The necessity of condition variables to constitute factors influencing the duration of emergency medical facilities is tested first. If a condition is always present in the outcome, it is necessary for the outcome to arise. The relationship between the necessity and sufficiency of the condition and outcome variables is determined by consistency and coverage. Consistency measures the extent to which the cases involved in the study can be explained by a single condition variable. Coverage, on the other hand, represents the reliability of interpretation. When the consistency is >0.9, the condition variable is necessary for the occurrence of the outcome.

The consistency of the seven condition variables was <0.9 (Table 3), and no single condition variable explained the outcome variable effectively. This indicates that the construction duration of emergency medical facilities is affected by a combination of conditions, rather than being the result of the action of a single condition. It is necessary to explore the joint synergistic effect among the variables by starting from the combined effect at the level of the seven condition variables.

**Table 3:** Necessary conditions analysis (China, 2021).

**Principal factors**	**Variable name**	**Consistency**	**Coverage**
Project-scope	NHB	0.614092	0.648741
	CF	0.856283	0.727342
Project-complexity	LPC	0.616714	0.574778
Material-condition	LBR	0.493741	0.661054
Manage-attributes	CTM	0.520506	0.674807
	IM	0.456187	0.588511
Other factors	CPAW	0.651228	0.575000

The consistency of the construction form of the condition variable was 0.856, which does not constitute a necessary condition alone but has high explanatory power. The simpler the construction form, the greater the duration advantages in public health emergencies. The consistencies of the condition variables of number of hospital beds, level of patient care, construction team management, and combination of peace and warfare were all >0.5, indicating that these condition variables could explain the outcome variables to some extent.

### Sufficient conditions

#### Parsimonious and intermediate solutions

The software is used to analyze all the variables in a group form to obtain solutions. The core and marginal conditions are distinguished by comparing the intermediate solution with the parsimonious solution, the conditions that appear in both the intermediate solution and the parsimonious solution are core conditions, and the conditions that appear only in the intermediate solution are peripheral conditions (32).

Parsimonious (33) means any remainder that will help generate a logically simpler solution is used, regardless of whether it constitutes an “easy” or a “difficult” counterfactual case. Intermediate (33) means that only remainders that are “easy” counterfactual cases are allowed to be incorporated into the solution. The designation of “easy” vs. “difficult” is based on user-supplied information regarding the connection between each causal condition and the outcome. The results of the parsimonious and intermediate solutions are shown in Table 4.

**Table 4:** Parsimonious and intermediate solutions for emergency medical facilities (China, 2021).

**Configurations**	**Raw coverage**	**Unique coverage**	**Consistency**
**Parsimonious solution**			
CF^*^~LBR^*^~IM^*^CPAW‘	0.451955	0.451955	0.856723
~LPC^*^CF	0.257511	0.016630	0.96
CF^*^IM^*^~CPAW	0.245146	0	0.989336
NHB^*^CF^*^IM	0.24088	0.01371	0.990198
Frequency cutoff	1		
Consistency cutoff	0.864286		
Solution coverage	0.723176		
Solution consistency	0.893373		
**Intermediate solution**			
LPC^*^CF^*^~LBR^*^~ CTM^*^~IM^*^CPAW	0.409633	0.193252	0.864403
NHB^*^LPC^*^CF^*^~LBR ^*^~IM^*^CPAW	0.237244	0.0208631	0.874725
NHB^*^~LPC^*^CF^*^~ LBR^*^~CTM^*^IM^*^~CPAW	0.169886	0.0685503	0.986159
NHB^*^~LPC^*^CF^*^LBR ^*^CTM^*^IM^*^~CPAW	0.137756	0.0364211	0.982986
Frequency cutoff	1		
Consistency cutoff	0.864286		
Solution coverage	0.636803		
Solution consistency	0.905262		

**Figure 2:**
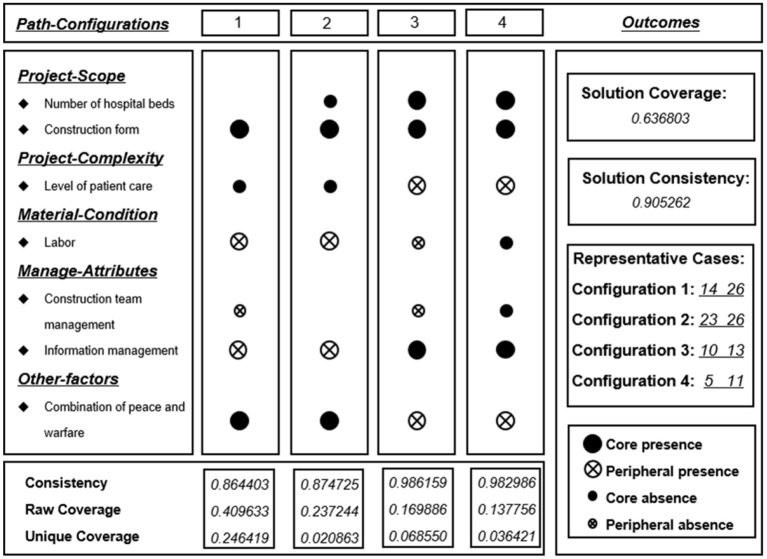
Sufficiency analysis for duration path-configurations including parsimonious and intermediate solutions (China, 2021).

Based on the solutions, the path configurations affecting the duration of emergency medical facilities are presented in Figure 2.

When the consistency is >0.8, it indicates that the cases represented in the grouping meet the requirements of the consistency test (34). The consistency of the combined results was 0.905, indicating that four antecedent condition groupings became sufficient conditions for the outcome variables. The coverage of the four configurations was 0.637, indicating that these four antecedent condition groupings covered ~63.7% of the cases of emergency medical facilities.

#### Path-configuration analysis

##### Configuration 1

The core conditions of this configuration were simple construction form, no combination of peace and warfare, less labor, and no use of information management, and the peripheral conditions were mild patient class and fewer managers. The consistency was 0.864 and the original coverage was 0.4096, indicating that the results of this configuration covered 40.96% of the case scenarios. This path can well explain the ability of the shelter hospital to rapidly complete the mass treatment of patients with mild COVID-19 in a very short period of time during a public health emergency. The shelter hospital in the representative construction case renovates stadiums or expo centers, which is a good method and path for quickly treating mildly ill patients in public health emergencies due to the simple structural form, which does not require a great deal of labor and management personnel.

##### Configuration 2

The raw coverage of this configuration was 0.2372, which is similar to that of configuration 1, except that the number of hospital beds in the construction cases in configuration 2 is smaller. This shows that there is less need for a construction management team when constructing this kind of shelter hospital, which highlights the superiority of the shelter hospital construction form. The experience of this small-scale hospital construction can give project managers a broader choice in project decision-making and management.

##### Configuration 3

The raw coverage of this configuration was 0.1699. Unlike configurations 1 and 2, which mainly focus on the path of duration control in a shelter hospital, configuration 3 explains how to better control the duration of a public health hospital facility that admits patients with severe COVID-19. The approaches adopted in the representative cases are all optimized and upgraded on the functions of the old hospital and are aided by information technology. This configuration not only has a smaller construction volume and lower requirements for labor and management personnel but also uses the combination of peace and warfare, which has significant advantages in resource savings.

##### Configuration 4

The raw coverage of this configuration was 0.1378. Configuration 4 is the same as configuration 3, except for the difference in the amount of labor and managers. It is clear that this configuration mainly differs in terms of the number of hospital beds. Under the same external conditions, a larger building size also requires more managers and more labor to complete the construction task. When comparing configurations 3 and 4, it can be observed that the renovation of a facility is more advantageous than a new construction in terms of construction time and resource saving, but the overall number of beds for patients with severe COVID-19 is less.

#### Robustness tests

A robustness check analysis was performed to verify the robustness of the results. The method changes the thresholds of the calculation process and analyzes whether the grouping results produce significant changes. The raw consistency threshold was adjusted from 0.8 to 0.85, and the PRI value was adjusted from 0.75 to 0.8. The calculation showed that the before and after results of the generated variables' histogram results on the interpretation of the outcome variables were consistent. The grouping results obtained from the analysis and calculation in the previous section were robust.

## Discussion

### Main findings

The construction process of public health emergency medical facilities in 30 different cities in China was analyzed, and six factors affecting the construction duration of these facilities were identified (Table 5). The construction duration was not independently affected by a single condition variable but by multiple influencing factors.

**Table 5:** Factors affecting the construction duration of emergency medical facilities (China, 2021).

**Classes**	**Specific factors**
Project-scope	Construction costs, gross floor area, number of hospital beds, construction form, project contract management, variations
Project-complexity	Client's attributes, site conditions/site access, buildability of design, level of patient care, quality of design co-ordination, quality management
Material-condition	Labor, materials, machinery, medical equipment
Manage-attributes	Design team management, construction team management, communication management for decision, information management, productivity
Environment	Natural environment, economic, epidemic environment, social political environment
Other-factors	Combination of peace and warfare, patriotism

Different planning and design strategies and project management methods should be adopted for different levels of patient care to ensure that public health emergency medical facilities are completed as planned. Some strategies and recommendations for duration management are presented.

First, the construction of emergency medical facilities should focus on planning and design and the selection of a reasonable construction form (35). The greater the number of hospital beds and the higher the level of patient care, the greater the scale of work and the higher the requirements for project management. The simpler the construction form, the shorter the construction duration. The more reasonable form of construction should be given priority under the premise of satisfying the medical function. The characteristics of several common construction forms of medical facilities are listed in Table 6.

**Table 6:** Characteristics of different public health emergency medical facilities (China, 2021).

**Construction form**	**Medical functions**	**Construction days**	**Number of hospital beds**	**Representative cases**	
Prefabricated buildings	Severe illnesses	Larger	Shorter	Case 2	Case 3
				Case 6	Case 8
Hospital conversion	Severe illnesses	Lesser	Short	Case 10	Case 11
Large buildings conversion	Mild illnesses	Larger	Short	Case 16	Case 17
				Case 22	Case 29

Second, coordination and management should be strengthened, and labor and other resources should be reasonably deployed. The shortage of labor resources and materials needs to be focused on, and these resources need to be organized early in the construction process. A lack of efficient project management will also make project construction difficult. Therefore, well-qualified management personnel and efficient management are key factors for the success of public health emergency medical facilities.

Third, information technology should be vigorously used to improve schedule management. During the response to the public health event of COVID-19, BIM technology greatly benefited the construction of different projects. Its main advantages are as follows: first, it can visually and graphically analyze the options; second, it can realize efficient node optimization and pipeline collision testing to reduce the difficulty of complex hospital systems; and third, it can simulate and analyze the outdoor wind environment to reduce the possibility of infections in healthcare workers.

Finally, the concept of a “combination of peace and warfare” means that a hospital that usually provides general medical services will quickly change its function to become a specialized hospital for infectious diseases and other epidemics during public health emergencies. This concept saves resources and has significant advantages in terms of construction time.

## Limitations

There are still some areas that can be improved in the current study. First, although the comparative analysis of the selection of conditional variables was as careful as possible, it still contained some subjectivity. Second, when we extracted data from 30 construction cases and assigned variables, although the data came from different website platforms for comparison and measurement, there may still have been data errors, which may have affected the necessity analysis and configuration effect analysis of a single variable. Finally, the application of QCA in configuration research can analyze the configuration of most cases, but individual cases with unclear characteristics will be ignored. Although this defect had limited influence on the purpose of this study, it will make the final research conclusion and the practice during the construction period not wholly consistent.

## Conclusion

It is usually assumed that shortening the construction duration of public health emergency medical facilities requires only a significant investment in labor and materials. However, this study finds that the main factors affecting construction duration are the scope of the project, the complexity of the project, the material situation, the project environment, and the project management attributes. Meanwhile, construction duration is not independently influenced by a single influencing factor but is the result of multiple factors. One category is for treating patients with mild COVID-19 using the construction of a simple form of a square cabin hospital. The simple form of a square cabin hospital required only a small input in management and labor resources. The other category is for treating severe COVID-19 using the hospital reconstruction method, which has more advantages than new containers and boarding houses, and information management tools have improved project management efficiency. At the same time, the combination of flat and epidemic conditions can achieve hospital savings regarding resources. This study can provide experience and a reference for the establishment of standard specifications for the construction of public health emergency medical facilities and efficient management of similar projects.

## Data availability statement

The original contributions presented in the study are included in the article/supplementary material, further inquiries can be directed to the corresponding author.

## Author contributions

WZ: conceptualization, formal analysis, and writing—review and editing. HH: collected and curation and visualization. Both authors have read, review, and approved the final manuscript.

## References

[1] Ferstad O Gu A Lee RY Thapa I Shin AY Salomon JA. A model to forecast regional demand for COVID-19 related hospital beds. MedRxiv. (2020). 10.1101/2020.03.26.20044842

[2] Castro MC de Carvalho LR Chin T Kahn R França GV Macário EM. Demand for hospitalization services for COVID-19 patients in Brazil. MedRxiv. (2020). 10.1101/2020.03.30.20047662

[3] Emanuel EJ Persad G Upshur R Thome B Parker M Glickman A. Fair allocation of scarce medical resources in the time of Covid-19. New Engl J Med. (2020) 382:2049–55. 10.1056/NEJMsb2005114 32202722

[4] Litton E Bucci T Chavan S Ho YY Holley A Howard G. Surge capacity of Australian intensive care units associated with COVID-19 admissions. Med Aust. (2020) 212:463–7. 10.5694/mja2.50596 32306408 PMC7264562

[5] Suter P M. Good rules for ICU admission allow a fair allocation of resources, even in a pandemic. Swiss Med Wkly. (2020) 150:w20230. 10.4414/smw.2020.20230 32208496

[6] Legido-Quigley H Asgari N Teo YY Leung GM Oshitani H Fukuda K. Are high-performing health systems resilient against the COVID-19 epidemic? Lancet. (2020) 395:848–50. 10.1016/S0140-6736(20)30551-1 32151326 PMC7124523

[7] White DB Lo B. A framework for rationing ventilators and critical care beds during the COVID-19 pandemic. AMA. (2020) 323:1773–4. 10.1001/jama.2020.5046 32219367

[8] Lazzerini M Barbi E Apicella A Marchetti F Cardinale F Trobia G. Delayed access or provision of care in Italy resulting from fear of COVID-19. Lancet Child Adolesc Health. (2020) 4:e10–1. 10.1016/S2352-4642(20)30108-5 32278365 PMC7146704

[9] Kong X Guo C Lin Z Duan S He J Ren Y. Experimental study on the control effect of different ventilation systems on fine particles in a simulated hospital ward. Sustain Cities Soc. (2021) 73:103102. 10.1016/j.scs.2021.103102 34189016 PMC8222082

[10] Cai Y Huang T Liu X Xu G. The effects of “Fangcang, Huoshenshan, and Leishenshan” hospitals and temperature on the mortality of COVID-19. MedRxiv. (2020). 10.1101/2020.02.26.20028472 32742816 PMC7380280

[11] Chen L-K Yuan R-P Ji X-J Lu X-Y Xiao J Tao J-B. Modular composite building in urgent emergency engineering projects: a case study of accelerated design and construction of Wuhan Thunder God Mountain/Leishenshan hospital to COVID-19 pandemic. Autom Const. (2021) 124:103555. 10.1016/j.autcon.2021.103555 34803228 PMC8589358

[12] Feng Z-H Cheng Y-R Ye L Zhou M-Y Wang M-W Chen J. Is home isolation appropriate for preventing the spread of COVID-19. Public Health. (2020) 183:4. 10.1016/j.puhe.2020.03.008 32388010 PMC7141470

[13] Ge X-Y Pu Y Liao C-H Huang W-F Zeng Q Zhou H. Evaluation of the exposure risk of SARS-CoV-2 in different hospital environment. Sustain Cities Soc. (2020) 61:102413. 10.1016/j.scs.2020.102413 32834932 PMC7375302

[14] Chen S Zhang Z Yang J Wang J Zhai X Bärnighausen T. Fangcang shelter hospitals: a novel concept for responding to public health emergencies. Lancet. (2020) 395:1305–14. 10.1016/S0140-6736(20)30744-3 32247320 PMC7270591

[15] National Health Commission. Press Conference of the Point Prevention and Control Mechanism of the State Council on April 7 2020. Beijing: National Health Commission (2020).

[16] Zhu W Wang Y Xiao K Zhang H Tian Y Clifford SP. Establishing and managing a temporary coronavirus disease 2019 specialty hospital in Wuhan, China. Anesthesiology. (2020) 132:1339–45. 10.1097/ALN.0000000000003299 32195700 PMC7155903

[17] Luo H Liu Li C Chen K Zhang M. Ultra-rapid delivery of specialty field hospitals to combat COVID-19: lessons learned from the Leishenshan Hospital project in Wuhan. Autom Const. (2020) 119:103345. 10.1016/j.autcon.2020.103345 33311856 PMC7334964

[18] Megahed NA Ghoneim EM. Antivirus-built environment: lessons learned from Covid-19 pandemic. Sustain Cities Soc. (2020) 61:102350. 10.1016/j.scs.2020.102350 32834930 PMC7313520

[19] Li Y Lu Y Cui Q Han Y. Organizational behavior in megaprojects: integrative review and directions for future research. J Manag Eng. (2019) 35:04019009. 10.1061/(ASCE)ME.1943-5479.000069129515898

[20] Chang-Richards Y Rapp R Wilkinson S Von Meding J Haigh R. Disaster recovery project management: a critical service. Int J Project Manag. (2017) 35:783–7. 10.1016/j.ijproman.2017.03.003

[21] Dickens B L Koo R Wilder-Smith A Cook AR. Institutional, not home-based, isolation could contain the COVID-19 outbreak. Lancet. (2020) 395:1541–2. 10.1016/S0140-6736(20)31016-3 32423581 PMC7190294

[22] Zhou PH Zhang JJ Xie H Li HB Pu Y. Construction planning and planning management practice of Huoshenshan Hospital. Construct Technol. (2020) 49:25–9. 10.7672/sgjs2020120025

[23] Chan APC Scott D Chan APL. Factors affecting the success of a construction project. J Const Eng Manag. (2004) 130:153–5. 10.1061/(ASCE)0733-9364(2004)130:1(153)

[24] Chan DWM Kumaraswamy MM. Compressing construction durations: lessons learned from Hong Kong building projects. Int J Project Manag. (2002) 20:23–35. 10.1016/S0263-7863(00)00032-6

[25] Xu P Sun CY. Analysis on the influencing factors of post-Wenchuan Earthquake reconstruction Project duration. World Sci Technol Res Dev. (2008) 30:509–15.

[26] Woodside AG. Moving beyond multiple regression analysis to algorithms: calling for adoption of a paradigm shift from symmetric to asymmetric thinking in data analysis and crafting theory. J Bus Res. (2013) 66:463–72. 10.1016/j.jbusres.2012.12.021

[27] Fiss PC. A set-theoretic approach to organizational configurations. Acad Manag Rev. (2007) 32:1180–98. 10.5465/amr.2007.2658609229357889

[28] Ragin CC. Redesigning Social Inquiry: Fuzzy Sets and Beyond: Chicago, IL: University of Chicago Press (2009); Morgan SL. Redesigning social inquiry: fuzzy sets and beyond. Soc Force. (2010) 88:1936–8.

[29] Ordanini A Parasuraman A Rubera G. When the recipe is more important than the ingredients: a qualitative comparative analysis (QCA) of service innovation configurations. J Serv Res. (2014) 17:134–49. 10.1177/1094670513513337

[30] Greckhamer T. Cross-cultural differences in compensation level and inequality across occupations: a set-theoretic analysis. Organ Stud. (2011) 32:85–115. 10.1177/0170840610380806

[31] Barton H Beynon MJ. Do the citizens of Europe trust their police?. Int J Emerg Serv. (2015) 4:65–85.

[32] Rihoux B Ragin CC. Configurational Comparative Methods: Qualitative Comparative Analysis (QCA) and Related Techniques. London: Sage Publications (2008).

[33] Ragin CC Strand SI Rubinson C. User's guide to fuzzy-set/qualitative comparative analysis. Univ Arizona. (2008) 87:1–87.

[34] Fiss PC. Building better causal theories: a fuzzy set approach to typologies in organization research. Acad Manag J. (2011) 54:393–420. 10.5465/amj.2011.60263120

[35] Li Lin W Zhang Y Wang Z Moud HI. Effectiveness of prefabricated construction in major public health emergency management: a fsQCA Analysis. In: Proceedings of the 25th International Symposium on Advancement of Construction Management and Real Estate. Springer Singapore. (2021). p. 1163–73.

